# Disentangling metabolic impairment in the liver-heart axis: tissue-specific insulin sensitivity in type 2 diabetes

**DOI:** 10.3389/fendo.2026.1786303

**Published:** 2026-03-19

**Authors:** Queralt Martín-Saladich, Andreea Ciudin, Azahara Palomar, Cristina Gámez-Cenzano, Rafael Simó, Miguel A. González Ballester, J. Raul Herance

**Affiliations:** 1 Medical Molecular Imaging Research Group, Vall d’Hebron Research Institute (VHIR), Nuclear Medicine, Radiology and Cardiology Departments, Vall d’Hebron University Hospital (VHUH), Autonomous University Barcelona (UAB), Barcelona, Spain; 2 BCN MedTech, Information and Communication Technologies Department, Pompeu Fabra University, Barcelona, Spain; 3 Diabetes and Metabolism Research Group, VHIR, Department of Endocrinology, VHUH, UAB, Barcelona, Spain; 4 Centro de Investigación Biomédica en Red de Diabetes y Enfermedades Metabólicas Asociadas (CIBERDEM) (Instituto de Salud Carlos III), Madrid, Spain; 5 Catalan Institution for Research and Advanced Studies ICREA, Barcelona, Spain; 6 Centro de Investigación Biomédica en Red en Bioingeniería, Biomateriales y Nanomedicina (CIBERBBN) (Instituto de Salud Carlos III), Madrid, Spain

**Keywords:** cardiometabolic risk, hepatic disease, insulin resistance, liver-heart axis, type 2 diabetes

## Abstract

**Aims/hypothesis:**

The liver-heart axis in type 2 diabetes (T2D) reflects key metabolic interactions disrupted by insulin resistance (IR). Organ-specific effects of insulin remain unclear due to challenges in measuring tissue-level IR. This study aims to define liver-heart phenotypes and their associated metabolic impairments, which relate to hepatic fat accumulation linked to metabolic dysfunction-associated steatotic liver disease (MASLD) and coronary artery calcifications (CACs) tied to cardiovascular disease (CVD).

**Methods:**

In this cross-sectional study, 41 individuals with controlled T2D underwent biochemical tests and [18F]FDG PET/CT scans before and after a hyperinsulinemic euglycemic clamp (HEC). Tissue-specific insulin-mediated glucose uptake was derived from PET imaging, while CT provided data on radiodensity, volume, fat, and calcifications.

**Results:**

A strong inverse correlation was observed between myocardial and liver ΔSUV (r=-0.74, p=2×10^-7^), thus suggesting the liver-heart metabolic axis in T2D. Three phenotypes were determined according to increased risks of T2D comorbidities including MASLD and CVD: HepGluc[+]+mIR (high risk of MASLD and CVD), HepGluc[−]+mIR (high risk of CVD, low risk of MASLD), and HepGluc[−]+mIS (low risk of MASLD and CVD). Moreover, HOMA-IR was only reflective of organ-level dysfunction in HepGluc[+]+mIR, which was the most at-risk phenotype in terms of systemic and tissue-specific metabolic impairment, including higher inflammation, IR, liver fat, CACs, and biomarkers of MASLD and CVD.

**Conclusions/interpretation:**

This study explores a potential liver-heart metabolic axis in T2D, linked to insulin-mediated dysfunction that may originate in the heart and extend to the liver. The coexistence of organ-specific phenotypes suggests three possible risk profiles, with the HepGluc[+]+mIR phenotype appearing most consistent with advanced T2D progression. Careful identification of this phenotype could support improved monitoring and more personalized treatment strategies in T2D.

## Introduction

The liver-heart axis has been recently proposed in T2D due to the metabolic interactions and insulin-glucose dynamics between both organs (1, 2). For instance, insulin contributes to glucose uptake in the cells of cardiac tissue while also promoting triglyceride synthesis in the liver while suppressing glucose production (3). However, under conditions of insulin resistance (IR), this regulation is impaired, resulting in elevated blood glucose and in adipose tissue (AT) build-up in the liver, which can lead to metabolic dysfunction-associated steatotic liver disease (MASLD) (4). Therefore, insulin plays a fundamental role in both organs and could be a driver of a metabolic axis between them. Additionally, insulin has been also proposed to play a detrimental role by promoting smooth vascular cell differentiation into osteocyte-like cells, which contributes to atherosclerotic plaque formation in the vessels such as coronary artery calcium (CAC) and therefore to increased risk of cardiovascular disease (CVD) as well (5).

The existence of metabolic interactions between the heart and the liver has been proposed to play an important role in hepatic and cardiac tissue dysfunction, also known as the liver-heart axis (1, 2). For instance, previous works have targeted the relationship between CVD and MASLD (6, 7), which can evolve into metabolic dysfunction-associated steatohepatitis (MASH) due to fibrotic tissue (8). However, no studies have addressed the role this axis plays in the pathogenesis of T2D in terms of tissue-specific glucose uptake and IR, nor have they approached myocardial and hepatic IR-related consequences according to CVD and MASLD risk factors in T2D, respectively.

In addition, one of the main problems in T2D characterization is the lack of friendly methods to determine organ-specific metabolic affectation due to impaired insulin-glucose dynamics. Specifically, most studies have often used systemic indices including HOMA-IR, together with parameters used to evaluate T2D such as HbA1c or glucose plasma levels (9, 10). However, HOMA-IR does not always represent localized organ affectation (11). For instance, previous works have proposed the use of [^18^F]FDG PET imaging after a hyperglycemic euglycemic clamp (HEC) to assess tissue-specific insulin sensitivity (IS) – being inversely related to IR (12, 13).

Following such methodology, we have recently proposed the definition of myocardial and hepatic phenotypes associated to [^18^F]FDG uptake pre- and post-HEC on PET images, which simultaneously associate to different exposure to risk of developing CVD and MASLD due to IR, inflammation and fat accumulation. Thus, for the myocardium, phenotypes were defined as myocardial insulin-resistant (mIR) and myocardial insulin-sensitive (mIS) (11), whereas the liver classification included hepatic tissue without response to insulin (HepGluc[+]) and with response to insulin (HepGluc[−]) (14). In both cases, metabolically insulin impaired patients (mIR and HepGluc[+]) were found to be at higher risk due to CACs and hepatic steatosis relating to CVD and MASLD, respectively. In addition, alterations in the biochemical profile of the patients exhibited a tendency of further T2D progression in IR-affected phenotypes. Nonetheless, up until today no studies have addressed how these insulin-effect-based T2D phenotypes interact with each other, namely, how the heart-liver crosstalk associates to enhanced CVD and MASLD risk exposure according to biomarkers linked with the development of IR, CACs, and AT.

The goal of this study was to explore the relationship between the liver and heart in the context of organ-specific IR and AT, and their associated cardiovascular (CV) and MASLD risk factors. This included examining alterations in their biochemical profiles and assessing differences in MASLD and CVD progression based on previously published phenotypes linked to cardiac and hepatic disease risk. Thus, the aim of the present work was to untangle the connection between the liver and the heart in terms of systemic and organ-specific metabolic affectation in T2D.

## Methods

### Study design and overview

**Figure 1:**
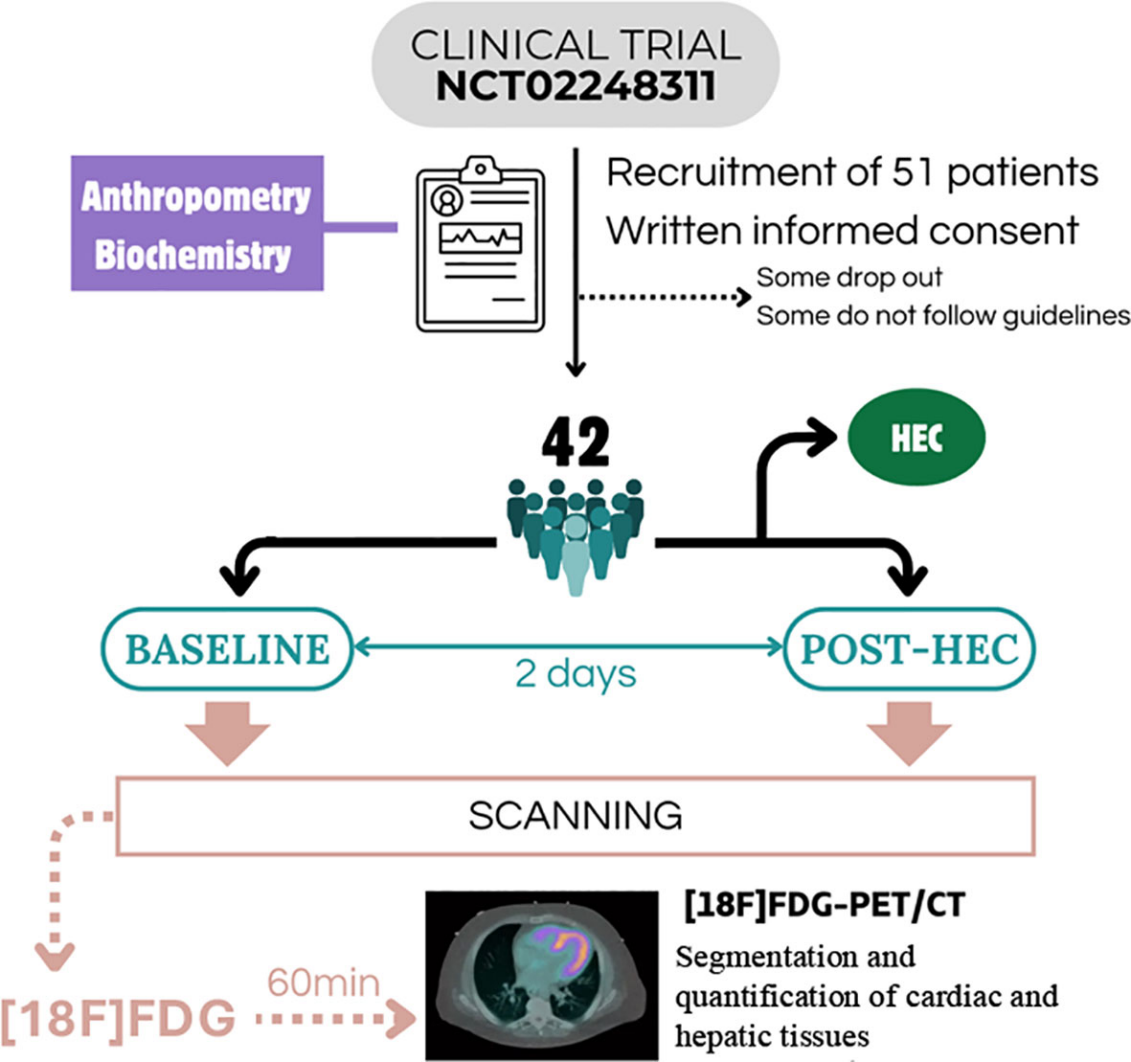
Flow diagram. Overview of participant recruitment, eligibility assessment, exclusions, and final sample included in the analyses. Biochemical data was obtained prior to the HEC procedure.

This analysis used the data from a pilot, single-center, proof-of-concept, cross-sectional clinical trial (ClinicalTrials.gov: NCT02248311) designed to evaluate tissue-specific IS in individuals with T2D using [18F]FDG PET/CT imaging integrated with HEC and supported by blood-sample biomarker alterations. Eligible participants underwent a standardized experimental workflow that included: (1) clinical, anthropometrical, and biochemical assessment, (2) whole-body [18F]FDG PET/CT imaging before and after HEC to assess tissue-specific IS, and (3) quantification of cardiometabolic risk indices. The full pipeline can be observed in **Figure 1**.

Blood samples and imaging data were collected under fasting conditions. Analysis of images were performed to determine insulin responses to glucose uptake by the myocardial and liver tissues.

All procedures adhered to the Declaration of Helsinki and were approved by the Ethics Committee of the Vall d’Hebron University Hospital (protocol PR(AG)01/2017). Moreover, written informed consent was obtained for all patients prior to any study activity.

### Patient characteristics

Eligible participants were adults aged 50–79 years with an established diagnosis of T2D for at least 5 years and stable metabolic control for at least one year prior to enrollment (fasting glucose <120 mg/dL and Hb1Ac <7.5%). Individuals were excluded if they had type 1 diabetes, a history of CV events, contraindications to PET/CT (including claustrophobia), comorbidities associated with limited life expectancy, high daily alcohol consumption (gender-adjusted thresholds), or active smoking unless abstinent for at least one year. These criteria were selected to minimize confounding by unstable or advanced cardiometabolic disease.

### Clinical and biochemical assessment

Fasting blood samples were obtained after overnight fasting conditions, with medication being withheld for 24 hours, and analyzed at the Biochemistry Core Facility from Vall d’Hebron Hospital using standardized clinical methods. Biochemical parameters were obtained relating to the glycemic and obesity control, lipid profile, liver enzymes, inflammatory markers, troponin I, and additional CVD and cardiometabolic risk markers.

HOMA-IR was calculated following the method proposed by Matthews et al. (9) Hepatic insulin clearance (HIC) was as estimated using the C-peptide-to-insulin molar ratio (15), since direct measurement or kinetic modeling data were not available in this pilot clinical trial.

### Imaging and hyperinsulinemic euglycemic clamp protocol

Two [^18^F]FDG-PET/CT images were acquired before and after the HEC, which was performed according to the specifications proposed by our group (11, 16) and others (13, 17). At least 8h of fasting and 24h of medication withdrawal were required before scanning sessions. The administered dose of [^18^F]FDG was of 1.9 MBq/Kg prior to image acquisition. At baseline conditions, a whole-body PET/CT scan was obtained 60 min after [^18^F]FDG injection and for a duration of 12 min. For the post-HEC scan, [^18^F]FDG was administered after the clamp protocol was stabilized. Images were acquired using a Siemens Biograph mCT 64S scanner (Siemens Healthcare, Erlangen, Germany) and were posteriorly reconstructed with a Gaussian filter (three iterations, and 21 subsets). Pixel spacing was 2.03642×2.03642 mm, matrix size was of 200×200 slice thickness was 3 mm. Comprehensive imaging details are available in previous publications (11, 16).

Whole-body IS (IS_HEC_) was assessed by taking the mean glucose infusion rate during the last 40 min, as proposed by Moreno-Navarrete et al. (17).

### Image analysis: tissue segmentation and quantification

To calculate the myocardial glucose metabolism, we segmented the tissue on both pre- and post-HEC images and obtained the glucose uptake data in terms of standardized uptake values (SUV). We then sub-tracted post-HEC minus baseline SUV (SUV_HEC_–SUV_baseline_) to obtain the ΔSUV, i.e., the insulin sensitivity (IS), of the region.

Myocardial glucose uptake was obtained using PMOD-PCARD (version 4.2) on PET scans according to the AHA-17 segmentation protocol. Epicardial adipose tissue (EAT) volume, attenuation and thickness values were assessed on CT images quantified in 3DSlicer. Attenuation values between -250 and -30 Hounsfield units (HU) were considered as AT, as previously described (18).

Liver segmentation was performed using multiple steps. First, nn-UNet was used to obtain the liver parenchyma segmentation mask on CT images. Then, 3DSlicer was used for registration and quantification purposes. After doing so, the mask was displayed over the registered PET image and quantification of the mask area was obtained. The same process was used for both baseline and HEC steps and the insulin-mediated effect on the hepatic was calculated following the same method as proposed for the myocardium. The percentage of fat in the liver was computed using our previously proposed algorithm in MATLAB (19), which analysed liver and spleen RD and volumes to calculate the number of fatty voxels per patient, and which suggested that the best criterion for fatty tissue in non-contrast enhanced CT was found to be liver-to-spleen RD difference <-10 HU (19).

Portal vein (PV) diameter was measured on axial CT image slices according to established guidelines, taking the maximum diameter as the distance between the outer vessel walls (20). Care was taken to ensure the measurement location was not at the confluence with nearby veins, such as the portal, splenic, and mesenteric veins, in order to avoid interference from diverging vessels.

All tissue segmentations were performed by a single experienced reader and reviewed by nuclear cardiology, radiology and nuclear medicine physicians as well as other imaging specialist investigators for accuracy. This approach ensured consistent and reliable quantification of epicardial adipose tissue and liver fat.

### CVD and MASLD risk indices

Multiple CV risk indices were also included in the present study were Framingham score (21), and CACs above 100 and 400 Agatston units (22). Biochemical features including troponin I (23), non-HDL cholesterol (24), TG/HDL (25) were also included. Lastly, ventricular ejection fraction (26) was evaluated as well.

Several MASLD risk indices were assessed including NAFLD liver fat score (NAFLD-LFS) (27), HEPAMET fibrosis score (HFS) (28), fatty liver index (FLI) (29), and hepatic steatosis index (HSI) (30). Liver stiffness measurements (LSM) according to FibroScan (31) were also evaluated.

### Phenotype definition

Two phenotypes in terms of myocardial IR, mIR and mIS, have been previously described by our group for patients with T2D (11), with mIR showing higher CV risk exposure due to IR, CACs, and alterations in their biochemical profile (11, 32). The definition of mIR and mIS was addressed by determining the ΔSUV by comparing the [18F]FDG-PET images before and after the HEC protocol. The grouping of patients was carried out by specialists in nuclear cardiology according to whether they visually observed an increase in myocardial glucose uptake after HEC or not. Thus, mIR related with insulin-resistant myocardium (tendency of ΔSUV ⪅ 0) and mIS for insulin-sensitive myocardium (tendency of ΔSUV ≥ 0).

Two other phenotypes have been proposed in terms of insulin-mediated hepatic glucose uptake for T2D as well according to HEC. Namely, HepGluc[−] for insulin response-related glucose uptake (tendency of ΔSUV < 0) and HepGluc[+] for non-insulin response-related glucose uptake (tendency of ΔSUV ≥ 0), respectively, and being HepGluc[+] exposed to higher MASLD risk and further progressed T2D rather than HepGluc[−] (14).

### Statistical analysis

For statistical analysis and plotting MATLAB R2023a (33) was used. Continuous variables were summarized as median [interquartile range] or mean ± standard deviation (SD), for non-parametric and parametric data, depending on the distribution assessed with the Shapiro-Wilk test. Categorical variables were reported as counts and percentages.

For the appraisal of the association between features, Spearman correlation coefficients (r) with two-tailed p-values were acquired. Differences between groups were computed by means of ANOVA (3-sample) for parametric data and Kruskal-Wallis (3-sample) analysis for non-parametric data. Post-hoc pairwise comparisons were conducted using Mann-Whitney U tests. Differences in categorical variables were assessed using Chi-square tests, with Fisher’s Exact tests being applied when counts were small (n<5). Effect sizes for categorical comparisons were estimated using Phi coefficients.

To control for multiple testing, the Benjamini-Hochberg false discovery rate (FDR) correction was applied to all p-values from post-hoc analyses. Adjusted p-values are reported alongside unadjusted values. Analyses were done for raw phenotype difference assessment and characterization, and no formal adjustment for potential confounders (e.g., age, sex, BMI) was applied.

All statistical tests were two-sided, and confidence intervals were set at 95% with p-values < 0.05. Graphical representations, including scatter plots with linear fits and bar plots with standard errors, were used to illustrate distributions and group differences.

## Results

Patient characteristics including anthropometrical, biochemical and IR measurements, CV risk indices, and MASLD scores are displayed in **Table 1**. Initial recruitment included fifty-one patients with T2D, although only forty-one were considered in the final analyses. Reason for exclusion of the final sample included withdrawal from the trial (n=5), and corrupted files or missing data (n=5).

**Table 1:** Patient characteristics.

Parameter	All (n=41)
Gender (M:F)	20:22
Age (years)	67 ± 7
BMI (kg/m^2^)	30.68 [28.44, 35.3]
Glucose (mg/dL)	120 [110, 145]
HbA1c (%)	7.3 [6.6, 7.65]
Neutrophiles (%)	62.05 [58.3, 65.8]
Lymphocytes (%)	25.75 [23.2, 30.9]
Hemoglobin (g/dL)	13.5 [12.2, 14.2]
Chloride (mmol/L)	103 [102, 105.5]
Protein (g/dL)	7.05 [6.8, 7.4]
IL-6 (pg/mL)	2.63 [1.4, 4.54]
AST (U/L)	23.5 [19, 33]
ALT (U/L)	21 [16, 35.5]
ALP (U/L)	73 [59.5, 95.5]
GGT (U/L)	27 [17.5, 45]
HDL (mg/dL)	46.5 [38, 52]
LDL (mg/dL)	93 [80, 118]
Cholesterol (mg/dL)	164.5 [148, 200]
FFA (mg/dL)	0.69 [0.58, 0.83]
TG (mg/dL)	111.5 [83, 176]
Insulin (mU/L)	16.38 [10.53, 26.21]
PIIINP (ng/mL)	6.96 [5.72, 9.23]
TIMP-1 (ng/mL)	266.3 [226.5, 311.2]
Hyaluronic acid (ng/mL)	42.48 [30.44, 81.52]
HOMA-IR	4.98 [3.23, 9.83]
IS_HEC_	2.35 [1.43, 4.68]
EF	68.5 [59, 76]
FS	21.6 [13.7, 30]
Myocardial ΔSUV	0.45 [0.12, 1.23]
EAT volume (cm3)	296.14 [248.87, 327.6]
EAT RD (HU)	-88.9 [-90.53, -85.8]
EAT thickness (mm)	11.51 [10.83, 13.13]
Liver ΔSUV	-0.01 [-0.17, 0.23]
HIC	4.66 [1.71, 7.57]
Liver fat %	33.49 [21.44, 72.98]
NAFLD-LFS	3.04 [1.78, 10.49]
FLI	0.9 [0.77, 0.97]
HSI	42.34 [40.26, 47.7]
HFS (%)	3.04 [1.78, 10.49]
FIB-4	1.73 [0.91, 3.24]
ELF	9.24 [8.54, 10.04]
LSM (kPa)	10.6 [5.4, 17.6]
PV diameter (mm)	20.85 [14.36, 24.31]
Spleen volume (cm^3^)	278.05 [211.01, 352.39]
Liver volume (cm^3^)	2231.17 [1628.05, 2606.19]
Spleen RD (HU)	34.01 [29, 37.74]
Liver RD (HU)	42.65 [18.09, 48.53]

Data indicated as median ± interquartile range [Q1, Q3], except for age that has been shown as median ± SD.

BMI, Body mass index; AST, Aspartate aminotransferase; ALT, Alanine aminostransferase; ALP, Alkaline aminostransferase; GGT, Gamma-glutamyl aminostransferase; HDL and LDL, High- and low-density lipoproteins; FFA, Free fatty acids; TG, Trigylcerides; IS_HEC_, Whole-body IS; EF, Ejection fraction; FS, Framingham score; EAT, Epicardial adipose tissue; HIC, Hepatic insulin clearance; NALFD-LFS, NAFLD Liver fat score; FLI, Fatty liver index; HSI, Hepatic steatosis index; HFS, HEPAMET fibrosis score; FIB-4, Fibrosis index 4; ELF, Enhanced liver fibrosis score; LSM, Liver stiffness measurement; PV, Portal vein; RD, Radiodensity; HU, Hounsfield units.

Insulin-glucose dynamics in the heart and the liver have allowed the identification of myocardial (mIR and mIS) and hepatic phenotypes (HepGluc[+] and HepGluc[−]). However, not all HepGluc[+] patients matched with mIR subjects or vice versa, for which we proposed a new classification according to myocardial and hepatic phenotype coexistence as it follows: HepGluc[+]+mIR (n=19). HepGluc[−]+mIS (n=16), and HepGluc[−]+mIR (n=6). The combination of HepGluc[+]+mIS was not observed in the sample (n=0). A highly significant association was observed (p = 9 × 10^-5^), with a large effect size (ϕ = 0.62). *Post-hoc* power analysis indicated that the current sample provided >99% power to detect this effect. The minimum sample required to achieve 80% power would have been 2 participants per group, and for 95% power, 3 participants per group. Therefore, despite the relatively small sample size, the study was adequately powered for this primary outcome of the proof-of-conecpt clinical trial.

**Figure 2:**
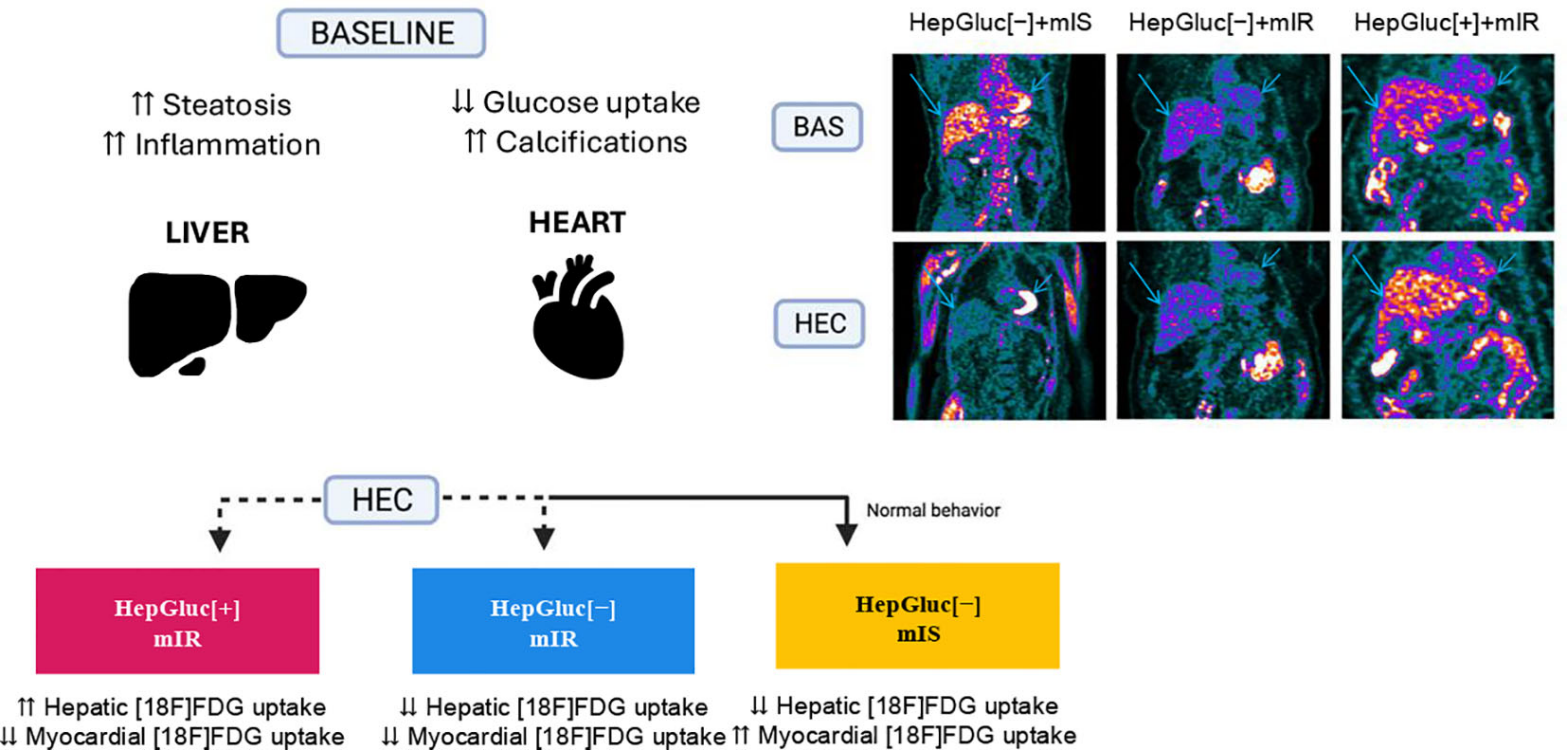
Characteristics of each phenotype. [18F]FDG uptake changes in the myocardium and liver that characterize the newly defined phenotypes: HepGluc[+]+mIR, HepGluc[−]+mIR, and HepGluc[−]+mIS. Blue arrows are in the liver and yellow arrows in the heart for reference.

A summary of the combination of phenotypes and their characteristics is displayed in **Figure 2**. Additional information on the effects of medication intake can be observed in **Supplementary Table 1**, which displayed no statistically significant differences among phenotypes.

**Figure 3:**
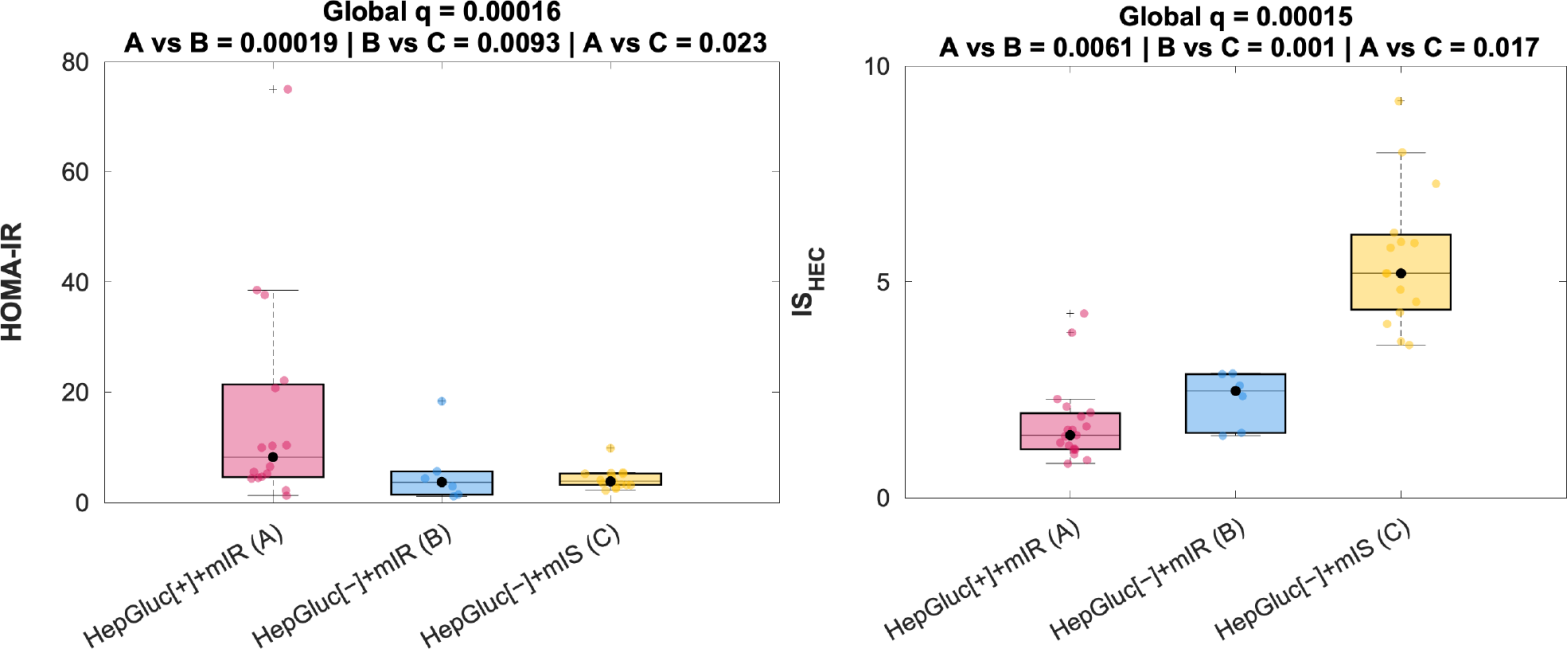
Systemic IR according to each phenotype. Distribution of systemic IR indices, HOMA-IR and IS_HEC_, according to each combination of heart and liver phenotypes. P-values are corrected for multiplicity.

As seen in **Figure 3**, systemic IR or IS parameters displayed significant trends of affectation between the newly defined phenotypes – considering HepGluc[−]+mIS phenotype as the least affected and HepGluc[+]+mIR as the most affected by systemic IR.

**Figure 4:**
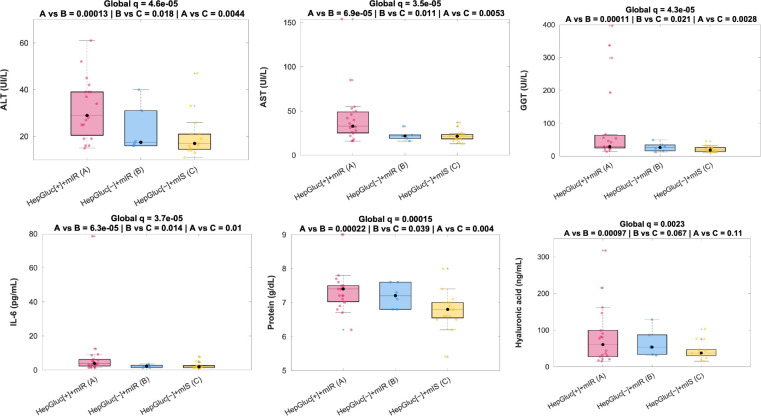
Biochemical alterations according to each phenotype. Distribution of blood parameters according to each combination of heart and liver phenotypes. ALT, Alanine aminotransferase; AST, Aspartate aminotransferase; GGT, Gamma-glutamyl transferase; IL-6, Interleukin 6. P-values are corrected for multiplicity.

In addition, some biochemical parameters showed a similar behavior, with altered features exhibited for the HepGluc[+]+mIR phenotype, which can be observed in **Supplementary Table 2**. A trend of higher ALT, AST, GGT, IL-6, hyaluronic acid, and protein was observed for HepGluc[+]+mIR, followed by HepGluc[−]+mIR, and eventually HepGluc[−]+mIS, as observed in **Figure 4**.

### The liver-heart axis: interacting organs

#### Insulin-mediated glucose uptake

**Figure 5:**
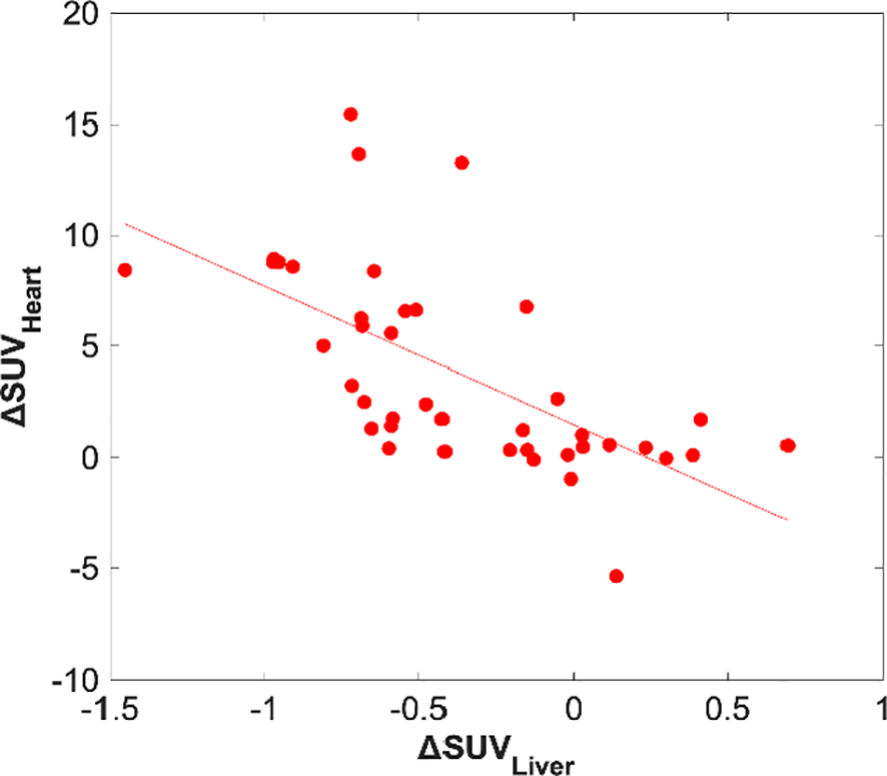
ΔSUV relationship between the heart and the liver. Scatter plot displaying the association between myocardial vs. hepatic glucose uptake with a least-squares fit (R^2^ = 0.40).

An analysis between the heart and the liver quantified ΔSUV was performed to assess the relationship between both organs in terms of response to insulin. Results showed a significative link (r=-0.74, p=2×10^-7^) between myocardial and hepatic ΔSUV, as seen in **Figure 5**. Moreover, the tendency of values between both organs has been observed to be inverse, thus increasing ΔSUV in the heart is associated with decreasing ΔSUV in the liver, and vice versa.

In terms of HOMA-IR, we compared it with the difference between the liver and heart ΔSUV, which displayed a significant relationship for the whole T2D population (r=0.45, p=0.006). Additionally, it did not show any statistically significant link for HepGluc[−]+mIS (r=-0.13, p=0.65), or HepGluc[−]+mIR (r=-0.54, p=0.30). Nonetheless, a linear significant association was found for HepGluc[+]+mIR patients, relating HOMA-IR as a function of ΔSUV_Liver_ – ΔSUV_Heart_ (r=0.63, p=0.015). In addition, we did a similar approach with IS_HEC_ and ΔSUV_Liver_ – ΔSUV_Heart_. Results displayed a significant negative link between studied parameters for all patients (r=-0.85, p<0.0005) and for HepGluc[+]+mIR (r=-0.61, p=0.006), although no significant relationships were observed for HepGluc[−]+mIS (r=-0.27, p=0.33) or HepGluc[−]+mIR (r=-0.31, p=0.56) either. As a result, only HepGluc[+]+mIR patients exhibited a close link for HOMA-IR and ΔSUV_Liver_ – ΔSUV_Heart_, as well as for IS_HEC_ and ΔSUV_Liver_ – ΔSUV_Heart._ The ΔSUV_Liver_ – ΔSUV_Heart_ parameter was chosen due to the compensatory mechanisms between the heart and the liver due to insulin-glucose homeostasis. All these associations can be found in **Supplementary Figure 1** and **Supplementary Figure 2**.

### The liver-heart metabolic axis: risk exposure

#### CV disease

CV risk exposure has been measured using multiple indicators including CACs above 400 AU, functional parameters such as Framingham score, ejection fraction, systolic and diastolic volume, as well as biomarkers including troponin I, non-HDL cholesterol and TG/HDL. As observed in **Table 2**, in terms of CACs, a clear trend of higher percentage of patients with coronary atherosclerotic plaque above 400 AU was observed for HepGluc[+]+mIR, followed by HepGluc[−]+mIR and HepGluc[−]+mIS.

**Table 2:** Patient CV risk indices according to phenotypes.

Parameter	HepGluc[+]+mIR	HepGluc[−]+mIR	HepGluc[−]+mIS	p (q-FDR)
Troponin I	8 [4, 12]	9.5 [8, 11]	7 [4, 9]	0.013 (0.014)
TG/HDL	3.5 [2.07, 5.97]	2.34 [2.04, 2.7]	1.94 [1.23, 3.09]	3.7e-05 (1.6e-04)
Non-HDL-c (mg/dL)	128 [104, 162]	137.5 [115, 140]	117 [91, 128.5]	1.7e-04 (3.2e-04)
EF	68.5 [59, 76]	71 [61, 79.5]	67 [60.5, 78]	7.3e-04 (9.5e-04)
FS	21.6 [13.7, 30]	14.65 [13.7, 30]	17.15 [12.7, 29.7]	4.3e-04 (6.1e-04)
EAT volume (cm^3^)	296.14 [248.87, 327.6]	225.08 [178.33, 257.01]	264.64 [215.21, 299.77]	2.4e-05 (1.7e-04)
EAT RD (HU)	-88.9 [-90.53, -85.8]	-86.24 [-87.54, -85.54]	-88.75 [-91.19, -86.18]	3.7e-05 (1.5e-04)
EAT thickness (mm)	11.51 [10.83, 13.13]	10.83 [9.39, 11.94]	11.18 [9.42, 12.16]	2.3e-04 (3.8e-04)
% patients CACs>100 AU	61.11%	60.00%	56.25%	0.09*
% patients CACs>400 AU	55.56%	40.00%	31.25%	0.048*

Data indicated as median ± interquartile range [Q1, Q3], except for the analysis of CACs, which has been exhibited as total % of patients. Raw and FDR-corrected p-values (q-FDR) are displayed.

HepGluc[+], post-HEC enhanced liver uptake phenotype; HepGluc[−], post-HEC non-enhanced liver uptake phenotype; mIR, Myocardial IR; mIS, Myocardial IS; TG, Triglycerides; HDL, High density lipoprotein; Non-HDL-c, non-HDL cholesterol; EF, Ejection fraction; FS, Framingham score; EAT, Epicardial adipose tissue; RD, Radiodensity; HU, Hounsfield units; CACs, Coronary artery calcifications; AU, Agatston units. *Correction was not necessary for two independent tests on CACs using 100 and 400 AU thresholds.

On the other hand, findings relating to cardiac function parameters displayed a different tendency of affectation. For instance, increased Framingham score and decreased ejection fraction were observed for HepGluc[−]+mIR instead. Such set of patients was followed by HepGluc[+]+mIR and HepGluc[−]+mIS phenotypes, respectively. Additionally, a common pattern was seen for EAT features as decreased EAT volume and thickness were observed for HepGluc[−]+mIR, followed by HepGluc[−]+mIS and eventually HepGluc[+]+mIR. In addition, EAT radiodensity showed a similar behavior, with higher values for HepGluc[−]+mIR, followed by HepGluc[−]+mIS and eventually HepGluc[+]+mIR.

Lastly, biomarker analysis revealed distinct patterns across phenotypes. For instance, HepGluc[−]+mIR exhibited slightly higher troponin I levels, along with higher non-HDL cholesterol, a recognized indicator of cardiovascular risk based on lipid profiles (24). In contrast, the HepGluc[+]+mIR phenotype exhibited higher TG/HDL ratios, a lipid marker that has been linked to increased risk of coronary artery disease. Overall, most biomarkers associated with elevated cardiovascular risk were higher in the HepGluc[−]+mIR group, whereas CACs indicated greater risk in the HepGluc[+]+mIR phenotype.

#### Hepatic disease

MASLD can be evaluated using established scores including NAFLD-LFS, HSI, and FLI. However, MASLD can evolve into MASH by fibrosis development (8) for which we also tested for HFS, FIB-4, ELF, and LSM, as seen in **Table 3**.

**Table 3:** Patient MASLD and MASH indices according to phenotypes.

Parameter	HepGluc[+]+mIR	HepGluc[−]+mIR	HepGluc[−]+mIS	p (q-FDR)
HIC	4.66 [1.71, 7.57]	5.95 [4.16, 10.99]	5.92 [1.99, 9.12]	1.5e-02 (1.5e-02)
Liver fat %	22.12 [12.69, 57.22]	19.17 [15.57, 35.19]	13.65 [7.89, 23.06]	2.2e-04 (3.9e-04)
NAFLD-LFS	3.04 [1.78, 10.49]	0.93 [0.82, 1.08]	1.05 [0.47, 1.75]	4.5e-05 (1.3e-04)
FLI	0.9 [0.77, 0.97]	0.61 [0.45, 0.69]	0.66 [0.46, 0.84]	4.4e-07 (2.7e-05)
HSI (%)	42.34 [40.26, 47.7]	39.68 [38.78, 41.85]	40.25 [36.58, 47.64]	2.7e-05 (1.7e-04)
HFS	0.46 [-1.22, 1.95]	-0.13 [-0.65, 0.73]	-0.14 [-1, 0.26]	0.2 (0.2)
FIB-4	1.73 [0.91, 3.24]	1.36 [1.05, 2.08]	1.19 [0.82, 1.53]	7.0e-05 (2.0e-04)
ELF	9.24 [8.54, 10.04]	9.23 [9.01, 9.86]	9.03 [8.74, 9.39]	2.0e-03 (2.2e-03)
LSM (kPa)	10.6 [5.4, 17.6]	8.65 [6.9, 10.4]	4.4 [3.2, 5.6]	1.3e-04 (2.6e-04)
PV diameter (mm)	12.07 [10.93, 15.22]	11.91 [10.14, 13.41]	11.17 [10.17, 12.84]	1.3e-04 (2.6e-04)
Spleen volume (cm^3^)	278.05 [211.01, 352.39]	316.5 [212.66, 375.02]	201.42 [135.02, 246.35]	2.4e-04 (3.9e-04)
Liver volume (cm^3^)	2231.2 [1628.1, 2606.2]	1753.6 [1382.6, 1935.6]	1315.0 [1222.1, 1512.0]	3.3e-06 (5.1e-05)
Spleen RD (HU)	34.01 [29, 37.74]	33.94 [30.16, 36.15]	34.76 [31.28, 36.57]	6.5e-04 (9.0e-04)
Liver RD (HU)	42.65 [18.09, 48.53]	39.06 [24.04, 51.46]	44.82 [37.48, 51.27]	8.4e-04 (1.1e-03)

Data indicated as median ± interquartile range [Q1, Q3]. Raw and FDR-corrected p-values (q-FDR) are displayed.

HepGluc[+], post-HEC enhanced liver uptake phenotype; HepGluc[−], post-HEC non-enhanced liver uptake phenotype; HIC, Hepatic insulin clearance; NALFD-LFS, NAFLD Liver fat score; FLI, Fatty liver index; HSI, Hepatic steatosis index; HFS, HEPAMET fibrosis score; FIB-4, Fibrosis index 4; ELF, Enhanced liver fibrosis score; LSM, Liver stiffness measurement; mIR, Myocardial IR; mIS, Myocardial IS; PV, Portal vein; RD, Radiodensity; HU, Hounsfield units.

For instance, steatosis indices including NAFLD-LFS, HSI and FLI exhibited the same trend affecting each phenotype, with bigger values for HepGluc[+]+mIR, followed by similar values for HepGluc[−]+mIR and HepGluc[−]+mIS. However, in terms of fibrosis, FIB-4 and LSM showed a clear pattern of higher values for the HepGluc[+]+mIR phenotype, followed by HepGluc[−]+mIR, and lower values for the HepGluc[−]+mIS phenotype, whereas no such behavior was observed for ELF or HFS.

On the other hand, results showed that increased percentage of fat in the liver, calculated by liver-to-spleen RD (19), as well as liver volume, were found for HepGluc[+]+mIR, followed by HepGluc[−]+mIR and eventually HepGluc[−]+mIS. Furthermore, PV hypertension was assessed by increased PV diameter and splenomegaly (34, 35), was exhibited for HepGluc[+]+mIR, followed by HepGluc[−]+mIR and HepGluc[−]+mIS.

Thus, further progressed MASLD was observed for HepGluc[+]+mIR in all cases, with a trend to higher fibrosis and PV hypertension as well. In addition, lower HIC was observed for the phenotype including HepGluc[+] when compared to the combinations using HepGluc[−].

## Discussion

The liver-heart axis has been recently described and has been one of the challenges of recent T2D research (2, 6, 7). The interconnections between both organs in terms of glucose homeostasis relying on adequate insulin signaling contribute to optimal functioning of the heart and the liver (36, 37). Impaired insulin sensitivity (IS) in T2D – also known as insulin resistance (IR) – has been associated with adverse outcomes, epicardial adipose tissue (EAT), increased coronary artery calcification (CAC), and features of metabolic dysfunction–associated steatotic liver disease (MASLD) (38–41). In this proof-of-concept, cross-sectional study, we explored potential interactions between myocardial and hepatic insulin-mediated glucose uptake patterns, integrating organ-specific fat accumulation and comorbidity profiles.

Previous work from our group has suggested myocardial (mIR, mIS) and hepatic (HepGluc[+], HepGluc[−]) phenotypes in T2D, with HepGluc[+] showing trends toward higher MASLD prevalence and mIR toward higher CAC scores (11, 14, 32). Using the data from patients included in this proof-of-concept clinical trial, the combination of liver and heart phenotypes allowed us to define only three preliminary liver–heart phenotypes: HepGluc[+]+mIR, HepGluc[−]+mIR, and HepGluc[−]+mIS. Notably, no participants exhibited the HepGluc[+]+mIS phenotype. This absence may reflect a biological pattern in which enhanced hepatic insulin-mediated glucose uptake rarely coexists with myocardial IR, suggesting that insulin dysfunction may emerge first in the myocardium and subsequently involve the liver. However, given the cross-sectional design and limited sample size, this observation remains hypothesis-generating and requires validation in larger, longitudinal cohorts. Importantly, these phenotypes should be viewed as dynamic metabolic states influenced by disease duration, glycemic control, and pharmacologic treatment rather than fixed traits.

Although there were no statistically significant differences in the use of antidiabetic or lipid-lowering medications across phenotypes, these therapies may nonetheless influence hepatic and myocardial glucose metabolism. Nevertheless, all imaging studies were performed under standardized conditions, including a medication withdrawal period of at least 24 hours prior to imaging. Moreover, the imaging protocol used to calculate the organ-specific IR, where each patient has their own baseline signal, significantly reduced any medication interference during our study. Hence the importance of performing two PET/CT scans per patient. Both PET scans were obtained under the same patient conditions, so the metabolic changes provide valuable information about insulin-glucose dynamics Therefore, the observed changes in glucose metabolism reflect intrinsic physiological differences across phenotypes and can still be meaningfully interpreted. Future studies with larger cohorts and stratification by medication type will be needed to further clarify these effects.

The three newly defined phenotypes differed substantially in insulin sensitivity and metabolic profiles. HepGluc[−]+mIS exhibited the highest insulin sensitivity as assessed by IS_HEC_, followed by HepGluc[−]+mIR, whereas HepGluc[+]+mIR showed the lowest insulin sensitivity. In contrast, HOMA-IR followed an inverse pattern, reflecting increasing systemic IR across phenotypes. Hepatic enzymes (AST, ALT, GGT) and additional biochemical markers, including IL-6 levels, hyaluronic acid, and total protein, were highest in HepGluc[+]+mIR, intermediate in HepGluc[−]+mIR, and lowest in HepGluc[−]+mIS. Collectively, these findings suggest distinct metabolic and inflammatory profiles across liver–heart phenotypes. However, given the small subgroup sizes and lack of longitudinal follow-up, these observations remain descriptive and cannot establish causality or prognostic significance, despite their consistency with known pathways of T2D progression and associated comorbidities (16, 42–47).

Differences in HOMA-IR and SUV-derived measures were also phenotype dependent. HepGluc[+]+mIR showed the strongest associations between HOMA-IR and ΔSUV differences, whereas no such relationships were observed in HepGluc[−]+mIR or HepGluc[−]+mIS. These findings suggest that the utility of HOMA-IR may vary according to organ-specific insulin responsiveness and should be interpreted cautiously. Trends in EAT volume, CAC burden, and liver fat percentage paralleled phenotypic severity of IR, collectively suggesting a higher likelihood of developing T2D-related comorbidities, including cardiovascular disease (CVD) and MASLD, particularly in the HepGluc[+]+mIR phenotype. These results support the potential value of three-way phenotyping in T2D, offering deeper insight into disease heterogeneity by capturing combined myocardial and hepatic dysfunction. Such an approach may be relevant for advancing personalized medicine strategies in T2D. We further hypothesize that HepGluc[−]+mIR represents an intermediate phenotype within a continuum of organ-specific insulin dysfunction.

The observed differences may reflect compensatory mechanisms between organs, including the liver’s buffering role in glucose metabolism (48), although this hypothesis requires confirmation in larger, longitudinal cohorts. HepGluc[+]+mIR participants exhibited particularly tight associations among HOMA-IR, IS_HEC_, and ΔSUV differences, whereas such relationships were absent in the other phenotypes. Our finding that HOMA-IR reflects organ-level dysfunction primarily in the HepGluc[+]+mIR phenotype underscores both the utility and limitations of systemic fasting-based indices. While HOMA-IR captures systemic IR, it may be influenced by HIC and compensatory myocardial glucose uptake, potentially underestimating or overestimating organ-specific dysfunction. In comparison, IS_HEC_ and imaging-derived measures provide a more direct assessments of hepatic and myocardial glucose metabolism. Together, these approaches highlight that HOMA-IR is most informative when interpreted in the context of organ-specific data.

Analysis of organ fat accumulation further reinforced these findings. HepGluc[+]+mIR participants exhibited the highest EAT volume and thickness, lowest EAT radiodensity, highest prevalence of CAC >400 AU, and highest liver fat content, consistent with prior associations between myocardial IR to CV risk (38) and hepatic insulin-mediated glucose uptake to MASLD (14). Although fibrosis indices did not differ significantly in liver-only phenotypes (14), the present study suggests that fibrosis-related alterations may emerge when hepatic and myocardial insulin dysfunction coexist. Specifically, higher fibrosis indices were observed in the HepGluc[+]+mIR phenotype, indicating a potential synergistic effect of dual-organ IR.

Evaluation of phenotype-specific cardiometabolic risk showed that HepGluc[+]+mIR participants exhibited the highest CVD and MASLD risk parameters, suggesting increased morbimortality driven by the coexistence of impaired cardiac and hepatic glucose metabolism. In contrast, HepGluc[−]+mIS participants displayed the lowest markers of IR, inflammation, and organ-specific alterations. Although exploratory, these gradients support the clinical relevance of identifying myocardial IR and hepatic glucose dysregulation. Such identification can be achieved using previously published non-invasive algorithms (14, 49) and may facilitate more precise and personalized management strategies in T2D.

Overall, the present data support the concept of a liver–heart axis in T2D and highlight the value of organ-specific phenotyping for understanding interactions between cardiac and hepatic insulin-mediated glucose metabolism, adiposity, and metabolic risk. However, the cross-sectional design limits causal inference, and longitudinal studies are required to confirm temporal relationships and clinical implications. These findings underscore the importance of accurately identifying individuals with the HepGluc[+]+mIR phenotype to optimize patient management and minimize the risks associated with misclassification or suboptimal treatment strategies. The strong linear association observed in this phenotype between HOMA-IR and the difference in hepatic versus myocardial ΔSUV suggests a potential role for combined imaging- and fasting-based metrics in identifying patients at elevated risk for CVD and MASLD.

### Strengths and limitations

This study integrates PET/CT imaging with and without HEC to explore tissue-specific insulin sensitivity in T2D, providing a novel organ-level perspective for liver and heart. Although no *a priori* sample size calculation was performed, the observed effect size and *post-hoc* power analysis indicate that the study was sufficiently powered to detect the main associations. Non-significant findings for other outcomes may reflect limited power for smaller effects; these results should be interpreted cautiously and confirmed in larger studies. This framing underscores the exploratory, proof-of-concept nature of the work. Overall, the findings highlight the interconnection between cardiac and hepatic alterations in individuals with T2D, supporting the concept of a liver–heart axis in the context of insulin–glucose dynamics.

Several subgroup analyses were conducted to explore phenotype-specific associations. However, some subgroups included a small number of participants, which limits the robustness of these findings. Therefore, some observed correlations should be considered preliminary, and differences between subgroups were not formally tested for statistical interaction. These trends require validation in larger, independent cohorts.

## Conclusions

In conclusion, a liver–heart axis exists in T2D, characterized by insulin-related disruptions in tissue-specific glucose metabolism and adipose tissue accumulation in both organs. The proposed cardio-hepatic phenotyping framework may improve assessment of disease extent and support more tailored patient management. Increased systemic insulin resistance and abnormal organ-specific insulin-mediated metabolic pathways were predominantly observed in the HepGluc[+]+mIR phenotype. HOMA-IR demonstrated potential utility primarily within this group, suggesting that fasting-based indices may better reflect insulin–glucose dynamics when both hepatic and myocardial responses are impaired.

Phenotype-specific differences were also evident in cardiometabolic risk markers: HepGluc[+]+mIR participants exhibited the highest CVD and MASLD risk, HepGluc[−]+mIR participants showed lower MASLD but elevated CVD risk, and HepGluc[−]+mIS participants displayed the lowest risk for both conditions. These findings suggest that transitions between liver–heart phenotypes – particularly from HepGluc[−]+mIR to HepGluc[+]+mIR – may represent stages in the progression of organ-specific insulin resistance and its comorbidities, warranting investigation in longitudinal studies.organ-specific IR and associated comorbidities, warranting further longitudinal study.

## Data Availability

The data analyzed in this study is subject to the following licenses/restrictions: The datasets generated during and/or analyzed in the current study are available from the corresponding author upon reasonable request. Requests to access these datasets should be directed to raul.herance@vhir.org.

## Glossary

[18F]FDG: F18 fluorodeoxyglucose
ΔSUV: Post-HEC −baseline SUV
ALT: Alanine aminotransferase
AST: Aspartate aminotransferase
BMI: Body mass index
CACs: Coronary artery calcifications
CaScore: Calcium score
CT: Computed tomography
CVD: Cardiovascular disease
EAT: Epicardial adipose tissue
ELF: Enhanced liver fibrosis
FFA: Free fatty acids
FIB-4: Fibrosis-4 score
FLI: Fatty liver index
GGT: Gamma-glutamyl aminotransferase
HA: Hyaluronic acid
HbA1c: Glycosylated haemoglobin
HDL and LDL: High- and low-density lipoproteins
HEC: Hyperinsulinemic euglycemic clamp
HepGluc[+]: Hepatic IR phenotype
HepGluc[−]: Hepatic IS phenotype
HFS: Hepamet fibrosis score
HIC: Hepatic insulin clearance
HOMA-IR: Homeostatic model assessment of IR
HIS: Hepatic steatosis index
HU: Hounsfield units
IL-6: Interleukin 6
IR: Insulin resistance
IS: Insulin sensitivity
IS_HEC_: Whole-body IS
LSM: Liver stiffness measurement
MASH: Metabolic-associated steatohepatitis
MASLD: Metabolic-associated steatotic liver disease
mIR: Myocardial IR phenotype
mIS: Myocardial IS phenotype
PET: Positron emission tomography
PIIINP: Type III Procollagen Peptide
PV: Portal vein
RD: Radiodensity
SFRP-1: Secreted frizzled-related protein 1
SUV: Standardized uptake value
T2D: Type 2 diabetes
TG: Triglycerides

## References

[1] El Hadi H Di Vincenzo A Vettor R Rossato M . Relationship between heart disease and liver disease: A two-way street. Cells. (2020) 9(3):567. doi: 10.3390/CELLS9030567, PMID: 32121065 PMC7140474

[2] Yan W Tao L Ma X . The liver and heart: How the two most beloved “babies” in the human body communicate. Cardiol Discov. (2021) 1:211–3. doi: 10.1097/CD9.0000000000000037, PMID: 41800202

[3] Giugliano D Ceriello A Esposito K . Glucose metabolism and hyperglycemia. Am J Clin Nutr. (2008) 87:217S–22S. doi: 10.1093/ajcn/87.1.217s, PMID: 18175761

[4] Hatting M Tavares CDJ Sharabi K Rines AK Puigserver P . Insulin regulation of gluconeogenesis. Ann N Y Acad Sci. (2018) 1411:21–35. doi: 10.1111/nyas.13435, PMID: 28868790 PMC5927596

[5] Fadini GP Pauletto P Avogaro A Rattazzi M . The good and the bad in the link between insulin resistance and vascular calcification. Atherosclerosis. (2007) 193:241–4. doi: 10.1016/J.ATHEROSCLEROSIS.2007.05.015, PMID: 17606264

[6] Ambale-Venkatesh B Lima JAC . Probing the liver-heart axis. Radiology. (2019) 291:338–9. doi: 10.1148/RADIOL.2019190264, PMID: 30835190

[7] Ismaiel A Dumitraşcu DL . Cardiovascular risk in fatty liver disease: The liver-heart axis—literature review. Front Med (Lausanne). (2019) 6:202. doi: 10.3389/FMED.2019.00202, PMID: 31616668 PMC6763690

[8] Wree A Broderick L Canbay A Hoffman HM Feldstein AE . From NAFLD to NASH to cirrhosis—new insights into disease mechanisms. Nat Rev Gastroenterol Hepatol. (2013) 10:627–36. doi: 10.1038/nrgastro.2013.149, PMID: 23958599

[9] Matthews DR Hosker JP Rudenski AS Naylor BA Treacher DF Turner RC . Homeostasis model assessment: Insulin resistance and β-cell function from fasting plasma glucose and insulin concentrations in man. Diabetologia. (1985) 28:412–09. doi: 10.1007/BF00280883, PMID: 3899825

[10] American Diabetes Association Classification and diagnosis of diabetes: Standards of medical care in diabetes-2020. Diabetes Care. (2020) 43:S14–31. doi: 10.2337/dc20-S002, PMID: 31862745

[11] Herance JR Simó R Velasquez MA Paun B García-Leon D Aparicio C . Phenotyping type 2 diabetes in terms of myocardial insulin resistance and its potential cardiovascular consequences: A new strategy based on 18 F-FDG PET/CT. J Pers Med. (2022) 12(1):30. doi: 10.3390/JPM12010030, PMID: 35055345 PMC8778238

[12] Tam CS Xie W Johnson WD Cefalu WT Redman LM Ravussin E . Defining insulin resistance from hyperinsulinemic-euglycemic clamps. Diabetes Care. (2012) 35:1605–10. 10.2337/dc11-2339 PMC3379600 22511259

[13] Gerber BL Ordoubadi FF Wijns W Vanoverschelde JLJ Knuuti MJ Janier M . Positron emission tomography using(18)F-fluoro-deoxyglucose and euglycaemic hyperinsulinaemic glucose clamp: Optimal criteria for the prediction of recovery of post-ischaemic left ventricular dysfunction. Results from the European Community Concerted Action Multicenter study on use of(18)F-fluoro-deoxyglucose positron emission tomography for the detection of myocardial viability. Eur Heart J. (2001) 22:1691–701. doi: 10.1053/EUHJ.2000.2585, PMID: 11511119

[14] Martín-Saladich Q Simó R Ciudin A Baguña J Ramirez- Serra C Aguadé-Bruix S . Phenotypic patterns of metabolic dysfunction-associated steatotic liver disease in type 2 diabetes: The impact of insulin. Eur J Clin Invest. (2025) 55(9):e70050. doi: 10.1111/eci.70050, PMID: 40251759 PMC12362049

[15] Piccinini F Bergman RN . The measurement of insulin clearance. Diabetes Care. (2020) 43:2296. doi: 10.2337/DC20-0750, PMID: 33972319 PMC8132327

[16] Herance JR Martín-Saladich Q Velásquez MA Hernandez C Aparicio C Ramirez-Serra C . Identification of myocardial insulin resistance by using liver tests: A simple approach for clinical practice. Int J Mol Sci. (2022) 23:8783. doi: 10.3390/IJMS23158783, PMID: 35955920 PMC9369008

[17] Moreno-Navarrete JM Novelle MG Catalán V Ortega F Moreno M Gomez-Ambrosi J . Insulin resistance modulates iron-related proteins in adipose tissue. Diabetes Care. (2014) 37:1092–100. doi: 10.2337/DC13-1602, PMID: 24496804

[18] Kitagawa T Nakamoto Y Fujii Y Sasaki K Tatsugami F Awai K . Relationship between coronary arterial 18F-sodium fluoride uptake and epicardial adipose tissue analyzed using computed tomography. Eur J Nucl Med Mol Imaging. (2020) 47:1746–56. doi: 10.1007/S00259-019-04675-Z, PMID: 31897585

[19] Martín-Saladich Q Pericàs JM Ciudin A Ramirez-Serra C Escobar M Rivera-Esteban J . Metabolic-associated fatty liver voxel-based quantification on CT images using a contrast adapted automatic tool. Med Image Anal. (2024) 95:103185. 38718716 10.1016/j.media.2024.103185

[20] Stamm ER Meier JM Pokharel SS Clark T Glueck DH Lind KE . Normal main portal vein diameter measured on CT is larger than the widely referenced upper limit of 13 mm. Abdom Radiol (NY). (2016) 41:1931–6. doi: 10.1007/S00261-016-0785-9, PMID: 27251734

[21] D’Agostino RB Vasan RS Pencina MJ Wolf PA Cobain M Massaro JM . General cardiovascular risk profile for use in primary care: The Framingham heart study. Circulation. (2008) 117:743–53. doi: 10.1161/CIRCULATIONAHA.107.699579, PMID: 18212285

[22] Greenland P Blaha MJ Budoff MJ Erbel R Watson KE . Coronary calcium score and cardiovascular risk. J Am Coll Cardiol. (2018) 72:434. doi: 10.1016/J.JACC.2018.05.027, PMID: 30025580 PMC6056023

[23] Huynh K . Troponin I in CVD risk prediction. Nat Rev Cardiol. (2019) 16:386–. doi: 10.1038/s41569-019-0212-3, PMID: 31092916

[24] Brunner FJ Waldeyer C Ojeda F Salomaa V Kee F Sans S . Application of non-HDL cholesterol for population-based cardiovascular risk stratification: Results from the multinational cardiovascular risk consortium. Lancet. (2019) 394:2173–83. doi: 10.1016/S0140-6736(19)32519-X, PMID: 31810609 PMC6913519

[25] Da Luz PL Favarato D Faria-Neto JR Lemos P Chagas ACP . High ratio of triglycerides to HDL-cholesterol predicts extensive coronary disease. Clinics (Sao Paulo). (2008) 63:427. doi: 10.1590/S1807-59322008000400003, PMID: 18719750 PMC2664115

[26] Shah S Segar MW Kondamudi N Ayers C Chandra A Matulevicius S . Supranormal left ventricular ejection fraction, stroke volume, and cardiovascular risk: Findings from population-based cohort studies. JACC Heart Fail. (2022) 10:583–94. doi: 10.1016/J.JCHF.2022.05.007, PMID: 35902163

[27] Kotronen A Peltonen M Hakkarainen A Sevastianova K Bergholm R Johansson LM . Prediction of non-alcoholic fatty liver disease and liver fat using metabolic and genetic factors. Gastroenterology. (2009) 137:865–72. doi: 10.1053/J.GASTRO.2009.06.005, PMID: 19524579

[28] Ampuero J Pais R Aller R Gallego-Durán R Crespo J García-Monzón C . Development and validation of Hepamet fibrosis scoring system-a simple, noninvasive test to identify patients with nonalcoholic fatty liver disease with advanced fibrosis. Clin Gastroenterol Hepatol. (2020) 18:216–25.e5. doi: 10.1016/J.CGH.2019.05.051, PMID: 31195161

[29] Bedogni G Bellentani S Miglioli L Masutti F Passalacqua M Castiglione A . The fatty liver index: A simple and accurate predictor of hepatic steatosis in the general population. BMC Gastroenterol. (2006) 6:1–7. doi: 10.1186/1471-230X-6-33/TABLES/3 17081293 PMC1636651

[30] Lee JH Kim D Kim HJ Lee CH Yang JI Kim W . Hepatic steatosis index: A simple screening tool reflecting nonalcoholic fatty liver disease. Dig Liver Dis. (2010) 42:503–8. doi: 10.1016/J.DLD.2009.08.002, PMID: 19766548

[31] Lallukka S Sädevirta S Kallio MT Luukkonen PK Zhou Y Hakkarainen A . Predictors of liver fat and stiffness in non-alcoholic fatty liver disease (NAFLD) – an 11-year prospective study. Sci Rep. (2017) 7:14561. doi: 10.1038/S41598-017-14706-0, PMID: 29109528 PMC5674024

[32] Martín-Saladich Q Simó R Aguadé-Bruix S Simó-Servat O Aparicio-Gómez C Hernández C . Insights into insulin resistance and calcification in the myocardium in type 2 diabetes: A coronary artery analysis. Int J Mol Sci. (2023) 24:3250. doi: 10.3390/IJMS24043250, PMID: 36834662 PMC9959651

[33] The MathWorks Inc MATLAB & Simulink. Available online at: https://uk.mathworks.com/products/matlab.html.

[34] Helgesson S Tarai S. Langner T Ahlström H Johansson L Kullberg J . Spleen volume is independently associated with non-alcoholic fatty liver disease, liver volume and liver fibrosis. Heliyon. (2024) 10:e28123. doi: 10.1016/J.HELIYON.2024.E28123, PMID: 38665588 PMC11043861

[35] Lee J Choi S Cho SH Yang H Sung PS Bae SH . The portal venous pulsatility index and main portal vein diameter as surrogate markers for liver fibrosis in nonalcoholic fatty liver disease and metabolic-dysfunction-associated steatotic liver disease. Diagnostics. (2024) 14:393. 38396432 10.3390/diagnostics14040393 PMC10888470

[36] Bertrand L Horman S Beauloye C Vanoverschelde JL . Insulin signalling in the heart. Cardiovasc Res. (2008) 79:238–48. doi: 10.1093/CVR/CVN093, PMID: 18390897

[37] Tanase DM Gosav EM Costea CF Ciocoiu M Lacatusu CM Maranduca MA . The intricate relationship between type 2 diabetes mellitus (T2DM), insulin resistance (IR), and nonalcoholic fatty liver disease (NAFLD). J Diabetes Res. (2020) 2020:3920196. doi: 10.1155/2020/3920196, PMID: 32832560 PMC7424491

[38] Lee YJ Wang CP Hsu HL Hung WC Yu TH Chen YH . Increased epicardial adipose tissue (EAT) volume in type 2 diabetes mellitus and association with metabolic syndrome and severity of coronary atherosclerosis. Clin Endocrinol (Oxf). (2009) 70:876–82. doi: 10.1111/J.1365-2265.2008.03411.X, PMID: 18778397

[39] Davis TME . Diabetes and metabolic dysfunction-associated fatty liver disease. Metabolism. (2021) 123:154868. doi: 10.1016/j.metabol.2021.154868, PMID: 34400217

[40] Stabley JN Towler DA . Arterial calcification in diabetes: Preclinical models and translational implications. Arterioscler Thromb Vasc Biol. (2017) 37:205. doi: 10.1161/ATVBAHA.116.306258, PMID: 28062508 PMC5480317

[41] Ormazabal V Nair S Elfeky O Aguayo C Salomon C Zuñiga FA . Association between insulin resistance and the development of cardiovascular disease. Cardiovasc Diabetol. (2018) 17:122. doi: 10.1186/s12933-018-0762-4, PMID: 30170598 PMC6119242

[42] Sanyal D Mukherjee P Raychaudhuri M Ghosh S Mukherjee S Chowdhury S . Profile of liver enzymes in non-alcoholic fatty liver disease in patients with impaired glucose tolerance and newly detected untreated type 2 diabetes. Indian J Endocrinol Metab. (2015) 19:597. doi: 10.4103/2230-8210.163172, PMID: 26425466 PMC4566337

[43] Islam S Rahman S Haque T Sumon AH Ahmed AM Ali N . Prevalence of elevated liver enzymes and its association with type 2 diabetes: A cross‐sectional study in Bangladeshi adults. Endocrinol Diabetes Metab. (2020) 3(2):e00116. doi: 10.1002/EDM2.116, PMID: 32318634 PMC7170449

[44] Assy N Schlesinger S Hussein O . Elevated plasma protein C levels correlate with the presence of fatty liver (NASH and NAFLD). Gut. (2005) 54:729. doi: 10.1136/GUT.2004.060251, PMID: 15831928 PMC1774506

[45] Meléndez GC McLarty JL Levick SP Du Y Janicki JS Brower GL . Interleukin 6 mediates myocardial fibrosis, concentric hypertrophy, and diastolic dysfunction in rats. Hypertension. (2010) 56:225–31. doi: 10.1161/HYPERTENSIONAHA.109.148635, PMID: 20606113 PMC2921860

[46] Rehman K Akash MSH Liaqat A Kamal S Qadir MI Rasul A . Role of interleukin-6 in development of insulin resistance and type 2 diabetes mellitus. Crit Rev Eukaryot Gene Expr. (2017) 27:229–36. doi: 10.1615/CritRevEukaryotGeneExpr.2017019712, PMID: 29199608

[47] Wieckowska A PapouChado BG Li ZZ Lopez R Zein NN Feldstein AE . Increased hepatic and circulating interleukin-6 levels in human nonalcoholic steatohepatitis. Am J Gastroenterol. (2008) 103:1372–9. doi: 10.1111/J.1572-0241.2007.01774.X, PMID: 18510618

[48] Xanthopoulos A Starling RC Kitai T Triposkiadis F . Heart failure and liver disease: Cardiohepatic interactions. JACC Heart Fail. (2019) 7:87–97. doi: 10.1016/J.JCHF.2018.10.007, PMID: 30553904

[49] Martín-Saladich Q Simó R Herance JR Ballester MAG . MIRA: Myocardial insulin resistance app for clinical practice. Comput Methods Programs BioMed. (2025) 263:108674. doi: 10.1016/J.CMPB.2025.108674, PMID: 39978140

