# Supplementary figures and images for: Disentangling metabolic impairment in the liver-heart axis: tissue-specific insulin sensitivity in type 2 diabetes
Source: Front Endocrinol (Lausanne). 2026 Mar 19;17:1786303. doi: 10.3389/fendo.2026.1786303 (PMC13043365; doi:10.3389/fendo.2026.1786303)

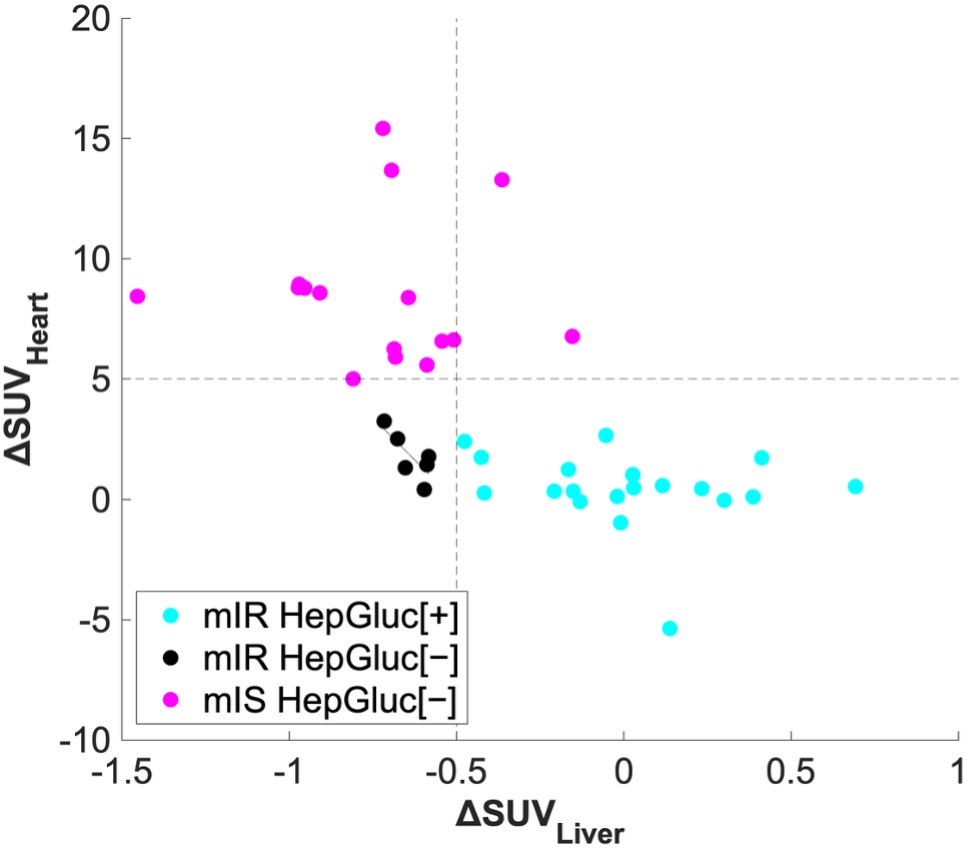

Supplement: Supplementary file 1 [file Image1.jpeg]

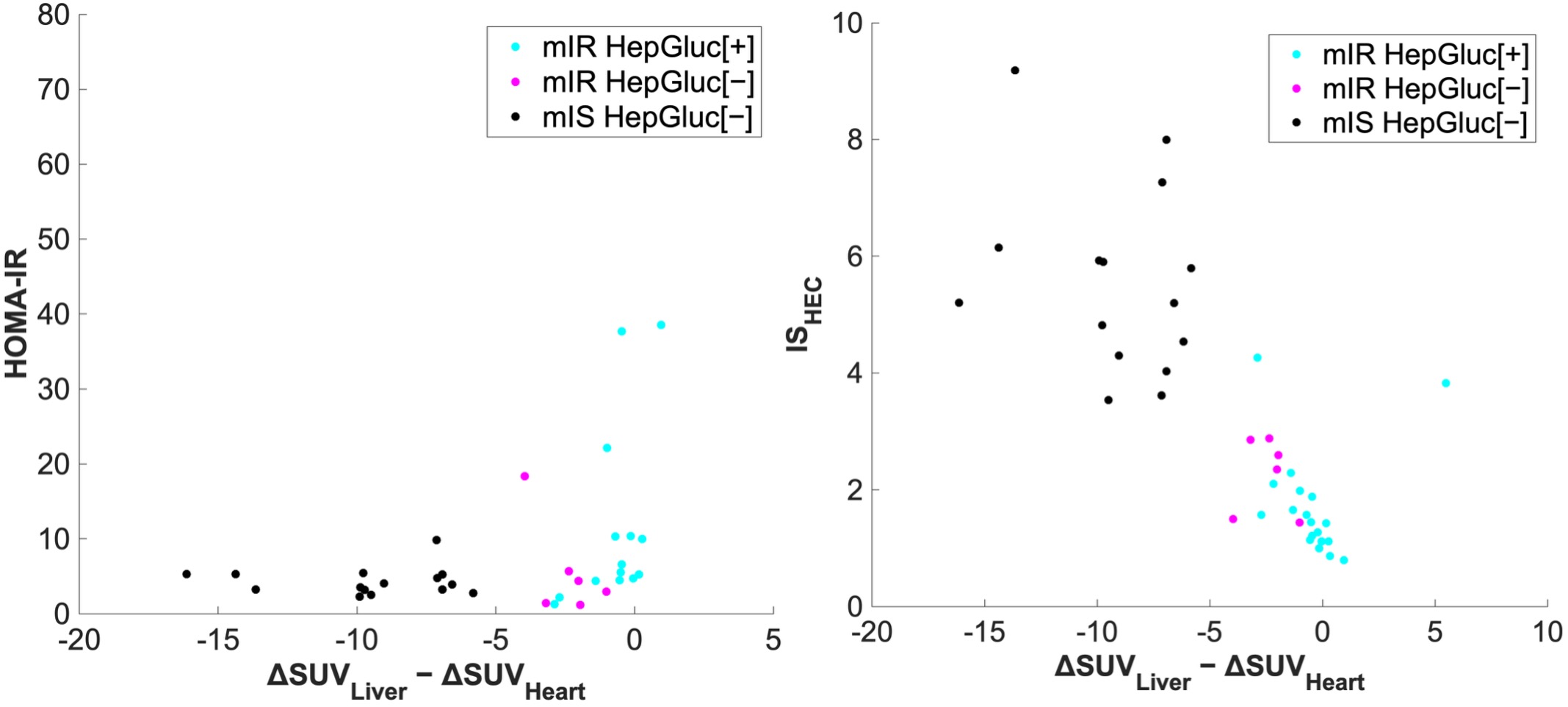

Supplement: Supplementary file 2 [file Image2.jpeg]
