# Supplementary material for: Disentangling metabolic impairment in the liver-heart axis: tissue-specific insulin sensitivity in type 2 diabetes
Source: Front Endocrinol (Lausanne). 2026 Mar 19;17:1786303. doi: 10.3389/fendo.2026.1786303 (PMC13043365; doi:10.3389/fendo.2026.1786303)
Supplement: Supplementary file 3 [file Table1.docx]

**Table S1. Differences in medication intake across liver-heart axis phenotypes.** The exhibited p-values were obtained from Chi-square tests on contingency tables. The number of patients with each medication intake and the corresponding % is displayed.

| **Medication type (n, %)** | **p-value** |
| --- | --- |
| Metformin/Pioglitazone (n=27, 66%) | 0.66 |
| Ca^2+^ channel blockers (n=6, 15%) | 0.49 |
| Ca^2+^ supplementation (n=3, 7%) | 0.52 |
| Vitamin D/Ca^2+^ usage helpers (n=7, 17%) | 0.27 |
| SGLT-2 inhibitors (n=15, 37%) | 0.76 |
| Insulin (n=21, 51%) | 0.65 |
| 𝛽-blockers (n=29, 71%) | 0.32 |
| Statins (n=27, 66%) | 0.66 |
| GLP-1 agonists (n=10, 24%) | 0.35 |
| Heparin (n=11, 27%) | 0.29 |
