# Supplementary material for: Disentangling metabolic impairment in the liver-heart axis: tissue-specific insulin sensitivity in type 2 diabetes
Source: Front Endocrinol (Lausanne). 2026 Mar 19;17:1786303. doi: 10.3389/fendo.2026.1786303 (PMC13043365; doi:10.3389/fendo.2026.1786303)
Supplement: Supplementary file 4 [file Table2.docx]

**Table S2. Biochemical and anthropometrical characteristics according to each phenotype.** Data indicated as median ± interquartile range [Q1, Q3]. Raw and corrected p-values are exhibited as well.

| **Parameter** | **HepGluc[+]+mIR (n=19)** | **HepGluc[−]+mIR (n=6)** | **HepGluc[−]+mIS (n=16)** | **p (q-FDR)** |
| --- | --- | --- | --- | --- |
| BMI (kg/m^2^) | 32.66 [30.69, 35.63] | 29.14 [28.28, 29.41] | 29.94 [27.77, 35.61] | 2.2e-06 (q=4.5e-05) |
| Glucose (mg/dL) | 122 [109, 160.5] | 115.5 [114, 132] | 122.5 [105, 143.5] | 0.00022 (q=0.00038) |
| HbA1c (%) | 7.4 [6.8, 8.2] | 7.6 [6.05, 8.2] | 6.9 [6.4, 7.4] | 4.4e-05 (q=0.00014) |
| Neutrophiles (×10^9^/L) | 4.3 [3.5, 5.2] | 4.5 [3.5, 5] | 4 [3.45, 5.8] | 0.0003 (q=0.00045) |
| Lymphocytes (×10^9^/L) | 1.8 [1.4, 2.3] | 1.7 [1.2, 2.2] | 1.85 [1.5, 2.75] | 0.00019 (q=0.00035) |
| Leukocytes (×10^9^/L) | 7.12 [5.7, 8.25] | 6.82 [5.52, 8.33] | 6.46 [5.78, 9.77] | 0.00026 (q=0.00041) |
| Platelets (×10^9^/L) | 257 [169, 317] | 207.5 [187, 225] | 287 [241, 339.5] | 8.7e-05 (q=0.0002) |
| Hemoglobin (g/dL) | 12.7 [11.8, 14] | 13.5 [12.65, 14.5] | 13.7 [12.55, 14.2] | 0.0027 (q=0.0029) |
| Chloride (mmol/L) | 102.5 [102, 105] | 102.5 [99, 104] | 104.5 [103, 109] | 0.00029 (q=0.00044) |
| Protein (g/dL) | 7.4 [7, 7.65] | 7.2 [6.8, 7.6] | 6.8 [6.5, 7] | 3.8e-05 (q=0.00015) |
| IL-6 (pg/mL) | 3.82 [2.18, 6.32] | 2.27 [1.4, 2.82] | 1.86 [1.4, 2.56] | 3.8e-06 (q=4e-05) |
| AST (U/L) | 33 [25, 50] | 22 [17.5, 28] | 21.5 [18.5, 23.5] | 1.2e-06 (q=3.8e-05) |
| ALT (U/L) | 29 [19, 39] | 17.5 [16, 31] | 17 [14, 23.5] | 5.6e-06 (q=4.9e-05) |
| ALP (U/L) | 80 [59, 99] | 67 [61, 93] | 73 [52, 92] | 0.00012 (q=0.00026) |
| GGT (U/L) | 29 [23, 66] | 26 [16, 34] | 18.5 [13.5, 26] | 3.7e-06 (q=4.6e-05) |
| HDL (mg/dL) | 44 [38, 52] | 47.5 [33, 55] | 49 [40, 52] | 0.00094 (q=0.0012) |
| LDL (mg/dL) | 102 [82, 111] | 115.5 [90, 123] | 86 [74.5, 104] | 0.0017 (q=0.002) |
| Cholesterol (mg/dL) | 170 [154, 209] | 189 [162, 200] | 161 [138, 178] | 0.00073 (q=0.00096) |
| FFA (mg/dL) | 0.75 [0.56, 0.85] | 0.78 [0.6, 0.98] | 0.63 [0.58, 0.78] | 0.002 (q=0.0023) |
| TG (mg/dL) | 154 [103, 203] | 110 [83, 127] | 96 [66, 144] | 3.3e-05 (q=0.00017) |
| Insulin (mU/L) | 21.77 [15.54, 74.83] | 11.51 [8.95, 13.5] | 15.12 [9.31, 19.24] | 3e-05 (q=0.00017) |
| PIIINP (ng/mL) | 6.27 [5.05, 9.69] | 7.04 [5.88, 8.68] | 7.64 [5.88, 9.16] | 0.0038 (q=0.004) |
| TIMP-1 (ng/mL) | 299.55 [219.9, 379.6] | 226.15 [214.7, 308.8] | 253.7 [230.9, 289.3] | 8e-05 (q=0.0002) |
| Hyaluronic acid (ng/mL) | 60.49 [27.49, 99.35] | 53.66 [33.78, 87.12] | 37.59 [30.21, 46.97] | 0.002 (q=0.0022) |
| HOMA-IR | 8.27 [4.61, 21.43] | 3.68 [1.44, 5.67] | 3.88 [3.19, 5.27] | 3.4e-05 (q=0.00016) |
| IS_HEC_ | 1.45 [1.12, 1.98] | 2.47 [1.5, 2.86] | 5.2 [4.3, 6.14] | 4e-05 (q=0.00015) |

Abbreviations: BMI, Body mass index; AST, Aspartate aminotransferase; ALT, Alanine aminostransferase; ALP, Alkaline aminostransferase; GGT, Gamma-glutamyl aminostransferase; HDL and LDL, High- and low-density lipoproteins; FFA, Free fatty acids; TG, Trigylcerides; PIIINP, Type III Procollagen Peptide; TIMP-1, Metallopeptidase Inhibitor 1; IS_HEC_, Whole-body IS.
